# Autoincudotomy as an uncommon etiology of conductive hearing loss: Case report and review of literature

**DOI:** 10.1016/j.radcr.2022.10.097

**Published:** 2023-02-01

**Authors:** Fathi Hilal, Jeffrey Liaw, Joseph P. Cousins, Arnaldo L. Rivera, Ayman Nada

**Affiliations:** a Department of Radiology, University of Missouri, Columbia, MO, USA; b Department of Otolaryngology, University of Missouri, Columbia, MO, USA

**Keywords:** Cholesteatoma, Autoincudotomy, CT temporal bone

## Abstract

Ossicular pathology is a recognized etiology of conductive hearing loss. Ossicular pathology includes 2 main categories, that is, ossicular chain fixation and ossicular discontinuity. Ossicular discontinuity can be congenital or acquired. Auto-incudotomy is an uncommon form of acquired ossicular discontinuity that usually occurs as a sequel of spontaneous expulsion of cholesteatoma. Typically, it manifests with conductive hearing loss without evidence of cholesteatoma. In this report, we presented CT imaging finding of a 34-year-old male with tympanic membrane perforation and defective long process of the incus (auto-incudotomy) with minimal middle ear granulation tissue and adhesions, sequela of cholesteatoma. Radiologists should pay attention for evaluation of ossicles especially in patients presented with conductive hearing loss.

## Introduction

The primary conduction apparatus for sound waves to the inner ear consists of the tympanic membrane and the ossicular chain [1]. The ossicular chain consists of 3 bones; the malleus, incus, and stapes joined by 2 synovial joints, the incudomalleolar and incudostapedial joints [2]. Loss of this conduction pathway would subsequently result in an inability to effectively transmit this sound wave and result in a conductive hearing loss (CHL).

Ossicular chain pathologies are a leading cause of CHL and can be divided into ossicular chain fixation (OF) or ossicular discontinuity (OD) [3]. Trauma can cause ossicular discontinuity and appears on high-resolution computed tomography (HRCT) as fractures of the malleus, incus or stapes, or as dislocation, for example, of the incudo-stapedial or incudo-malleolar joints [4]. Other examples of ossicular discontinuity caused by developmental agenesis of the ossicles [[5], [6], [7]]; erosion of the ossicles secondary to inflammation resulting in impaired bone remodeling, or acquired due to cholesteatoma [[8], [9], [10]].

Cholesteatomata can lead to ossicular erosions early in the course of the disease, most often affecting the long process of the incus [10]. Ossicular erosions dampen the acoustic transmission resulting in CHL [11]. Although erosion of any ossicle contributes to the CHL, erosion of the incus has the most significant effect on CHL [12].

Cholesteatomata can present in subtle ways with opacification of the Prussak's space [13]. Not uncommonly, they can extend beyond Prussak's space into the middle ear and mastoid, causing erosions of ossicles, scutum, facial nerve canal and mastoid antral walls. The destructive process can result in widening of the attic and the mastoid antrum [13,14]. In rare occasions, a cholesteatoma can slough, and spontaneously expulse [13,15]. In these cases, the sequala of osseous erosion will be evident, such as an air-filled space within the attic in the shape of the expulsed cholesteatoma, and/or erosion into mastoid air cells which result in auto-atticotomy and auto-mastoidectomy respectively [13,15,16]. An auto-mastoidectomy occurs late in the disease, requiring a larger cholesteatoma and more extensive osseous erosions compared to auto-atticotomy [13]. The same process can affect the ossicles which then have evidence of erosion without obvious associated soft tissue masses. Bayat et al. demonstrated that incus is the most commonly involved ossicle in chronic otomastoiditis being absent in 9.34% or eroded in 30.84% [17]. Gomaa et al. investigated involvement of the ossicles with different types of cholesteatomas and found that incus is the most involved ossicle (16/56 patients, 28.57%) [18]. Celebi et al. and Manasawala et al. investigated the incidence of spontaneous evacuation of secondary acquired cholesteatoma with the development of automastoidectomy and/or auto-atticotomy [15].

In this report, we present a case of CHL secondary to a spontaneously expulsed acquired cholesteatoma which complicated chronic otitis media. The disease resulted in erosion of the mastoid bone and the incudal long and lenticular processes, with replacement of a fibrous band in its place.

## Case presentation

A 34-year-old man with a past medical history significant for right chronic otitis media status post multiple myringotomy tube placements as a child presented with several years of right-sided conductive hearing loss and tinnitus, and intermittent ear pressure.

Otomicroscopy revealed an area of granulation tissue of the medial external auditory canal adjacent to the annulus of the right posterior tympanic membrane, with retraction of the posterior pars tensa just medial to this granulation tissue. The retraction pocket appeared extending towards the incudo-stapedial joint, concerning for involvement of the ossicles. The combination of the patient's history and physical findings were concerning for cholesteatoma. A high-resolution non-contrast CT scan (HR-NCCT) of the temporal bone was subsequently requested to better assess the middle ear and mastoid.

**Fig. 1:**
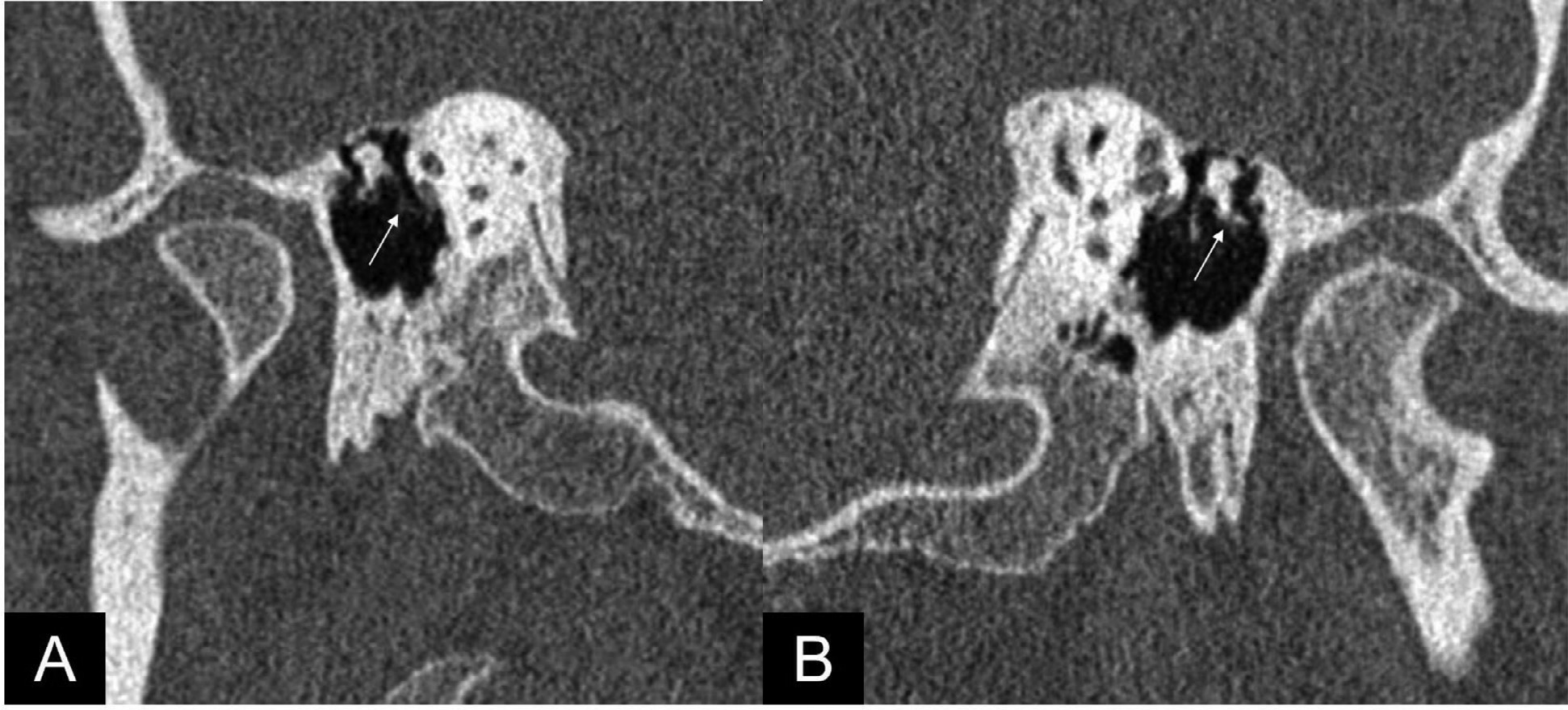
Autoincudotomy in a 34-year-old male presented with conductive hearing loss and otorrhea. High-resolution CT temporal bone with Poschl views (a) right and (b) left reveal loss of mineralization with defective long process of the incus (arrow) on the right side, compared to the left. Minimal hypodense granulation tissue and adhesions were observed.

**Fig. 2:**
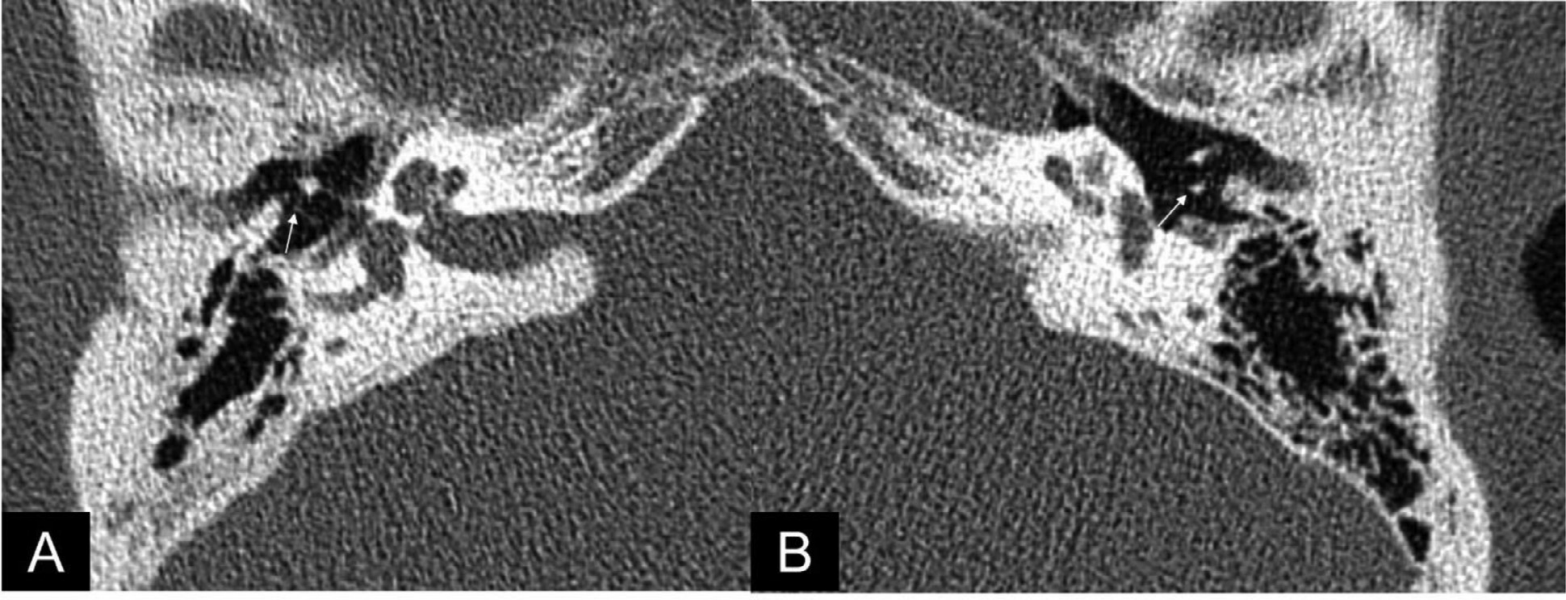
Autoincudotomy in a 34-year-old male presented with conductive hearing loss and otorrhea. High-resolution CT temporal bone with axial views (a) right and (b) left reveal loss of characteristic 2 osseous dots within the middle ear space, corresponding to demineralization and osseous defect of the long process of the incus on the right side, compared to the left (arrows).

A high resolution 0.6 mm isotropic HR-NCCT of the temporal bone revealed a hypoplastic right mastoid air cells with increased osseous density and small, mildly dysplastic ossicles. There was demineralization, resorption, and fibrous replacement of the distal long process of the incus near its incudo-stapedial articulation (Figs. 1 and 2).

Due to concern for cholesteatoma, the patient subsequently underwent a right middle ear exploration with a transcanal tympanoplasty. Evaluation of the ossicles confirmed erosion of the long process of the incus with adhesions around the stapes and chorda tympani. These adhesions were lysed with a Rosen needle. Palpation of the stapes confirmed normal mobility. The remnant incus was subsequently removed and a frisbee partial ossicular replacement prosthesis (PORP) was placed on the capitulum to reconstruct the ossicular chain and tragal cartilage graft placed lateral to this as a buttress. A CO_2_ laser was used to perform a myringoplasty to address the tympanic membrane retraction.

**Fig. 3:**
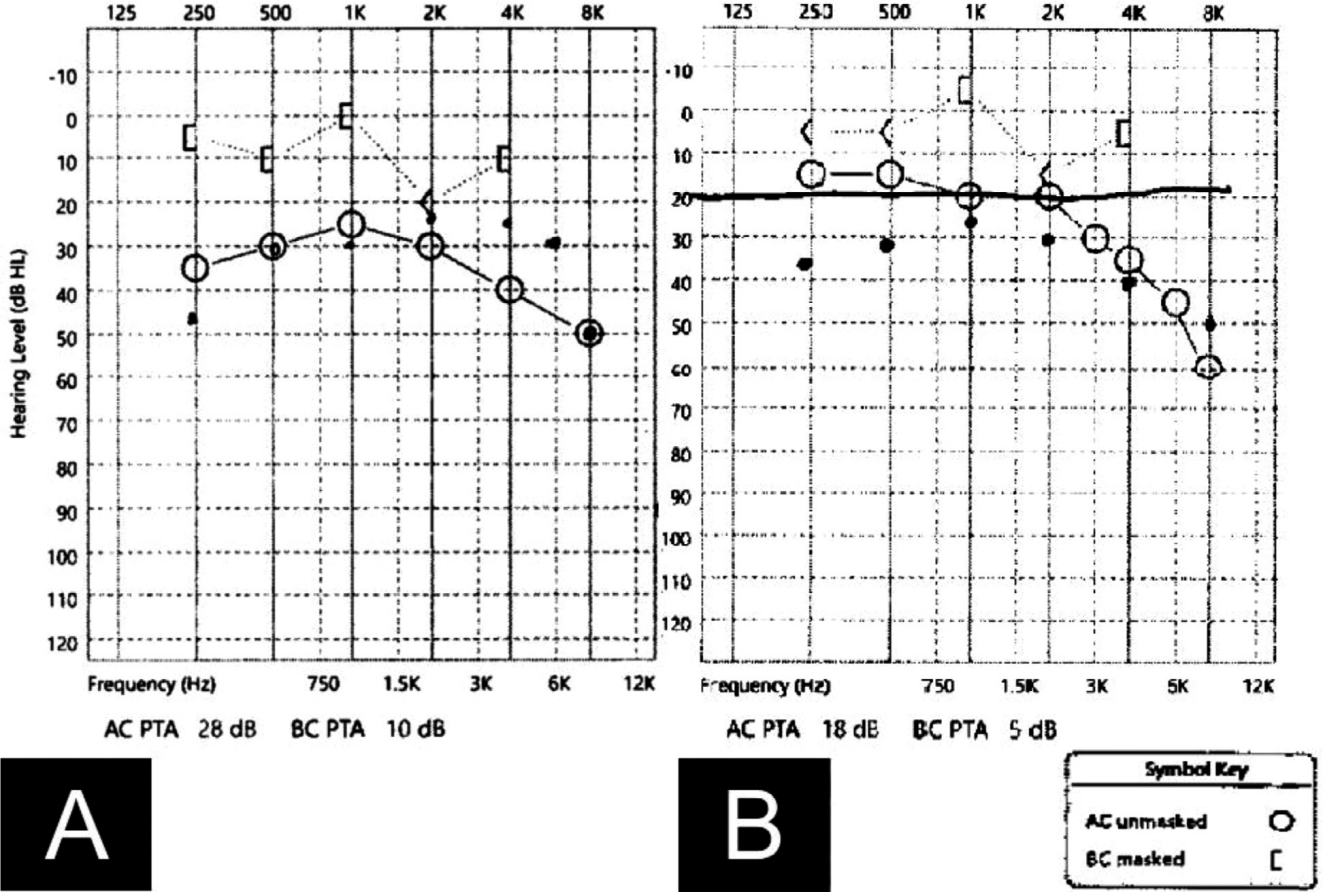
(a) Pre-operative and (b) post-operative audiograms demonstrating pure tone averages of the right ear and partial closure of the air bone gap with surgery.

Post-operatively, the patient was noted to have healed appropriately with no resultant perforation or granulation tissue. Post-operative audiogram demonstrated partial closure of the air bone gap in the right ear (Fig. 3).

## Discussion

Hearing loss associated with cholesteatomata is secondary to erosion of ossicles, especially the incus [10]. Other signs and symptoms include otorrhea, granulation tissue changes, ear pain, and tinnitus [19]. Our patient had a history of chronic otitis media which predisposed him to a cholesteatoma and presented with otorrhea, tinnitus, and CHL due to erosion of the incus.

In patients with CHL and a normal tympanic membrane on otoscopic examination, a high resolution NCCT of the temporal bone is a valuable tool as it can show the anatomical details of the middle ear cavity [20,21]. Cholesteatomata can appear on NCCT as a well-demarcated soft tissue, adjacent or surrounding the ossicles. Granulation tissue often appears to have smooth margins. Tympanic membrane retraction, loss of normal aeration within air-filled spaces of the tympanum, and variable erosions of the ossicles, tegmen tympani, and scutum are potential findings on NCCT and sequela of cholesteatoma and necessitate surgical intervention [13,15,22,23]. Osseous erosions are a feature that differentiates a cholesteatoma from chronic granulation tissue [15].

Magnetic resonance imaging (MRI) can play an important role in preoperative and postoperative evaluation of cholesteatomas. MRI signal intensities of cholesteatomata have a characteristic hypointense T1, hyperintense T2, with mild to moderate restricted diffusion on diffusion-weighted (DWI) sequences compared to the normal brain parenchyma [23]. DWI and delayed post contrast T1-weighted images have good sensitivity and specificity for detection of cholesteatomata, which appears as a non-enhancing soft tissue with restricted diffusion [24].

A spontaneously expulsed acquired cholesteatoma appears as an elliptical air cavity surrounded by granulation tissue on NCCT. The cavity can expand into the medial or lateral attic walls in patients with chronic ear discharge [15]. Prior imaging, in which a soft tissue mass was observed in the same location supports the diagnosis [15]. Subtle widening of lateral attic wall on axial NCCT images is another important clue in difficult cases where prior imaging is lacking [13]. In advanced cases, extensive destruction of mastoid air cells can result in a hypoplastic mastoid, that is, auto-mastoidectomy [15,16].

Absence of the incus is the most observed developmental middle ear anomaly [25]. It manifests as CHL at infancy with a normal ear drum and no history of middle ear infection [7,26]. Developmental CHL may be also associated with a stapes anomaly [3,25]. One study reported 3 cases with familial bilateral absence of the long process of the incus in 3 consecutive generations, that is, a grandmother, a mother and a daughter, and determined the mode of inheritance as autosomal dominant or X-linked [26]. Familial cases are commonly bilateral, whereas sporadic cases are unilateral [27].

Acquired resorption of the long and the lenticular processes can be caused by cholesteatomata or inflammation [9,10,28]. The incus is highly susceptible to injury because it floats in the middle ear space, undergoes continuous remodeling and resorption throughout life, and has relatively sparse deep blood supply [9,28,29]. Histological examination of distal incus has shown a rich network of blood vessels that run within the distal incus mucosa with anastomoses [30]. The lenticular process consists of a very thin pedicle, approximately 0.25 mm, and a thicker plate which connects to the stapes. This pedicle is very susceptible to resorption because of its thin diameter [30]. An absent long process may be replaced by a fibrous or a mucosal band [3,8]. Some acquired cases may not have an apparent etiology [31]. Advanced age is associated with loss of osteocytes and increased activity of osteoclasts which result in impaired bone remodeling and expedited resorption of the incus [30,32,33].

Our case demonstrates an individual with the sequela of cholesteatoma: autoincudotomy, tympanic membrane perforation, with subtle granulation tissue density, and adhesions. On surgical exploration, however, no cholesteatoma was noted. A spontaneously expulsed acquired cholesteatoma is suspected in this case.

HR-NCCT scan is a useful imaging tool for diagnosing ossicular abnormalities, for example, epitympanic fixation, ossicular malformation, discontinuity [4,8,9], and is better than MRI in delineating the ossicular chain [4,34]. Using a bone level with a window up to 300-500 Hounsfield units allows for better visualization of ossicular pathology [9]. Definitive diagnosis of an ossicular anomaly warrants exploratory tympanotomy [20,35].

Ossiculoplasty is the reconstruction of ossicles and can be performed by using either an autograft (eg, incus interposition graft), homograft (ie, from a cadaver) or most commonly from a synthetic materials such as titanium, hydroxyapatite and other materials [36]. Ossicular reconstruction using synthetic materials is done by either partial or complete ossicular replacement prosthesis [37]. The goal of this surgery is to re-establish the connection between the tympanic membrane and stapes, and close the air-bone gap on audiometry [37].

## Conclusion

Careful evaluation of the ossicles and tympanum on HR-NCCT of the temporal bone is essential in patients presenting with conductive hearing loss. Ossicular pathologies range from developmental absence, traumatic disruption, and resorption from aging, or due to cholesteatomata. Focal absence of an ossicle, most commonly the long process of incus, expansion of the tympanum, granulation tissue, and adhesions can be clues to for a spontaneously expulsed cholesteatoma.

## Patient consent

The authors have written informed consent for the publication of this case report.
